# Prevalence and factors related to sleep apnoea in ankylosing spondylitis

**DOI:** 10.1007/s10067-021-05924-z

**Published:** 2021-09-28

**Authors:** Adrian Wiginder, Carin Sahlin-Ingridsson, Mats Geijer, Anders Blomberg, Karl A. Franklin, Helena Forsblad-d’Elia

**Affiliations:** 1 Department of Public Health and Clinical Medicine, Section of Medicine, Umeå University, Umeå, Sweden; 2 Department of Radiology, Institute of Clinical Sciences, Sahlgrenska Academy, University of Gothenburg, Gothenburg, Sweden; 3 Department of Radiology, Sahlgrenska University Hospital, Gothenburg, Region Västra Götaland Sweden; 4 Faculty of Medicine, Lund University, Lund, Sweden; 5 Department of Surgical and Perioperative Sciences, Surgery, Umeå University, Umeå, Sweden; 6 Department of Rheumatology and Inflammation Research, Institute of Medicine, Sahlgrenska Academy, University of Gothenburg, Gothenburg, Sweden

**Keywords:** Ankylosing spondylitis, Observational study, Risk factors, Sleep apnoea

## Abstract

An increased prevalence of obstructive sleep apnoea (OSA) has been suggested in patients with ankylosing spondylitis (AS) in a few controlled studies. We aimed to study the prevalence of OSA compared to controls and to investigate if disease-related and non-disease-related factors were determinants of OSA in AS patients. One hundred and fifty-five patients with AS were included in the Backbone study, a cross-sectional study that investigates severity and comorbidities in AS. Controls were recruited from the Swedish CArdioPulmonary bioImage Study. To evaluate OSA, the participants were asked to undergo home sleep-monitoring during one night’s sleep. For each AS patient 45–70 years old, four controls were matched for sex, age, weight, and height. OSA was defined as an apnoea-hypopnoea index (AHI) ≥ 5 events/hour. Sixty-three patients with AS were examined with home sleep-monitoring, and 179 controls were matched with 46 patients, 45–70 years. Twenty-two out of 46 (47.8%) patients with AS vs. 91/179 (50.8%) controls had OSA (AHI ≥ 5 events/hour), *P* = 0.72. No differences in the sleep measurements were noted in AS patients vs. controls. In logistic regression analysis adjusted for age and sex, higher age, higher BMI, and lesser chest expansion were associated with the presence of OSA in the 63 AS patients. In the current study, patients with AS did not have a higher prevalence of OSA compared to matched controls. AS patients with OSA had higher BMI, were older, and had lesser chest expansion because of more severe AS compared to patients without OSA.

**Table:** 

Key points
• *Patients with ankylosing spondylitis did not have a higher prevalence of obstructive sleep apnoea versus matched controls.*
• *Patients with ankylosing spondylitis and obstructive sleep apnoea were older and had higher body mass index versus patients without obstructive sleep apnoea.*
• *Patients with ankylosing spondylitis and obstructive sleep apnoea had lesser chest expansion versus patients without obstructive sleep apnoea.*

**Supplementary Information:**

The online version contains supplementary material available at 10.1007/s10067-021-05924-z.

## Introduction

Ankylosing spondylitis (AS) is associated with an increased risk of several comorbidities [1]. Also, the patients often have sleeping problems, depending on pain, or on other causes [2] resulting in fatigue that may be a major problem for the patients but sometimes ignored [3, 4]. Obstructive sleep apnoea (OSA) is related to fatigue, and some small studies have suggested a link between AS and OSA [5, 6]. These studies found OSA to be more frequent in patients with AS compared to the reported prevalence in the general population [7, 8]. Recently, Walsh et al. reported the comorbidity burden using a large US administrative claims database and found the prevalence of OSA in AS patients to be higher versus controls (8.8% vs 5.1%, *P* < 0.001) [1]. We reported a similar OSA prevalence of 8.7% in a medical record-based study on AS patients in Northern Sweden [9]. Obstructive sleep apnoea is a condition with episodes of upper airway collapse, resulting in periods of apnoea and hypopnoea during sleep. The definition of OSA is a so-called apnoea-hypopnoea index (AHI) ≥ 5 events/h. When OSA is combined with significant daytime sleepiness evaluated by the Epworth sleepiness scale (ESS), the disorder is called OSA syndrome (OSAS) [10–12]. Furthermore, OSA is linked to obesity and cardio- and metabolic diseases and is therefore recognised as an important cause of morbidity and mortality [13].

The knowledge about OSA and OSAS in AS is scarce, and we therefore intended to study these conditions in well-characterised AS patients. We hypothesised that patients with AS have an increased prevalence of OSA compared with controls and that patients with more severe AS have an increased risk of OSA. The aims of this study were (1) to investigate the prevalence of OSA and explore the sleeping patterns in patients with AS compared to matched controls and (2) to determine factors associated with OSA/OSAS in AS patients.

## Materials and methods

### Patients and controls

Patients with AS, in Västerbotten County, Northern Sweden, between 18 and 70 years of age, without known dementia, with knowledge of the Swedish language, not being pregnant, and fulfilling criteria for AS [14] were asked to take part in the Backbone study that investigates comorbidities and severity of AS, previously described in detail [15]. The flow chart of the inclusion process of the 155 patients included in Backbone and in the current study is described in Supplementary Fig. 1. Spinal radiographic alterations were scored according to the modified Stoke Ankylosing Spondylitis Spinal Score (mSASSS) that ranges from 0 to 72 [17]. The Bath Ankylosing Disease Spondylitis Activity Index (BASDAI), Ankylosing Spondylitis Disease Activity Score with C-reactive protein (ASDAS-CRP), Bath Ankylosing Spondylitis Functional Index (BASFI), Bath Ankylosing Spondylitis Metrology Index (BASMI), and presence of metabolic syndrome [16] were assessed and chest expansion was measured at the 4th intercostal level [18]. High-sensitivity C-reactive protein (hsCRP), erythrocyte sedimentation rate (ESR), and blood lipids were analysed consecutively.

The control group comprised individuals in Västerbotten County from the nationwide Swedish CArdioPulmonary bioImage Study (SCAPIS). Overall, 30,000 men and women between 50 and 64 years of age, randomly selected from the Swedish population register, are included in SCAPIS [19]. It was voluntary both for the 155 patients with AS in the Backbone study and the 2,500 SCAPIS individuals from the Västerbotten County to evaluate signs of OSA. Sixty-three of the patients with AS and 1,011 of the SCAPIS individuals fully completed home sleep-monitoring (63/155, 40.6% vs 1011/2500, 40.4%; *P* = 0.97). Up to four controls (*n* = 179) to each of the 46 AS patients in the same age-span as SCAPIS individuals were matched for sex, age, weight, and length (Supplementary Fig. 1).

### Home sleep-monitoring device

The participants were examined during one night’s sleep with a home sleep-monitoring device (ApneaLink Air®, ResMed, CA, USA), which uses the same algorithm as its predecessor ApneaLink® [20]. Participants were provided with structured oral and the same written instruction on how to use the device. Apnoea is defined as a decrease in the airflow by ≥ 90% for at least 10 s and hypopnoea as a decrease in the airflow by > 30% for at least 10 s, combined with desaturation of ≥ 4% compared to the pre-event value. The frequency of obstructive sleeping events is reported as an Apnoea-Hypopnoea Index (AHI), the average number of apnoeas and hypopnoeas per hour of sleep. An AHI ≥ 5 events/h is defined as OSA and classified as mild (AHI ≥ 5 events/h and < 15 events/h), moderate (AHI ≥ 15 events/h and < 30 events/h), or severe (AHI ≥ 30 events/h) [11, 12].

### Sleep apnoea questionnaire

The participants answered the ESS [10] that rates (ranges 0–24) the risk of falling asleep in different everyday situations, and OSAS is defined as an AHI ≥ 5 events/h in combination with ESS ≥ 10 [11].

### Statistics

Results are expressed as mean ± standard deviation (SD), median (quartile 1 (Q1), quartile 3 (Q3)), or number and percentage (%), and comparisons between groups were performed with a *t*-test or the Mann–Whitney *U*-test as appropriate. Categorical parameters were compared with the Chi-square test or Fisher’s exact test. Based on a previous study [6], a power calculation was conducted showing that a sample size of at least 46 patients with AS and 184 controls would be needed to achieve a power of 80%, using a one-sided Fisher’s exact test at a 0.05 significance level. The matching of controls to the patients with AS was made by propensity score matching on sex, age, weight, and length. Univariable and age- and sex-adjusted logistic regression analyses were performed to identify variables associated with OSA. Odds ratios (OR) and 95% confidence intervals (CI) are given. Variables with *P* ≤ 0.05 in the comparative analyses were included in the logistic regression analyses. Statistics were performed using SPSS version 24 (SPSS Inc., IBM, Chicago, IL, USA). *P* ≤ 0.05 was considered statistically significant.

## Results

### Comparison between patients with AS and matched controls

The 46 patients with AS were slightly shorter and had somewhat higher BMI, whereas weight and sex distribution did not differ vs. controls (Table 1). The 46 patients displayed similar clinical characteristics to the rest of the 109 patients in Backbone (Supplementary Table 1). Out of these 46 patients, 22 had OSA vs. 91/179 controls, had OSA (47.8% vs 50.8%; *P* = 0.717). Eighteen (39.1%) AS patients had mild, four (8.7%) moderate, and none severe OSA, while 69 (38.5%) controls had mild, 17 (9.5%) moderate, and five (2.8%) severe OSA (*P* > 0.5).

**Table.1:** Comparison between 46 patients with ankylosing spondylitis (AS) and 179 matched controls included in the Swedish CArdioPulmonary bioImage Study (SCAPIS)

*Variables*	*Patients with AS* *n* = *46*	*SCAPIS-persons* *n* = *179*	*P-value*
General characteristics			
Male sex, n	30 (65.2)	123 (68.7)	0.65
Age, years	57.2 (7.5)	57.2 (4.5)	0.98
Weight, kg	81.1 (15.4)	79.5 (14.5)	0.56
Length, cm	169.8 (8.6)	172.3 (8.1)	0.031
BMI, kg/m^2^	28.1 (4.8)	26.6 (3.8)	0.025
Home sleep-monitoring			
AHI, events/h †	6.5 (6.4)	7.1 (7.4)	
	4.5 (2.0, 8.9)	4.9 (1.7, 9.9)	0.60
Apnoea Index, events/h †	2.3 (4.1)	3.0 (4.9)	
	1.0 (0.2, 2.8)	1.2, (0.3, 3.7)	0.36
Hypopnea index, events/h †	4.2 (4.4)	4.2 (4.4)	
	2.8 (0.9, 5.6)	2.7 (1.0, 5.7)	0.99
Average saturation, % O_2_	93.1 (1.7)	93.0 (1.6)	0.87
Lowest desaturation, % O_2_	84.9 (4.1)	85.2 (5.0)	0.79
Lowest saturation, % O_2_	83.7 (4.5)	83.4 (6.4)	0.75
Base saturation, % O_2_	95.4 (1.7)	95.4 (2.1)	0.91
Average pulse, beats per minute	62.3 (8.7)	61.9 (7.6)	0.76

Values are mean (SD) or number of patients (%) unless otherwise indicated

^†^Values are also given as median (quartile 1, quartile 3)

*BMI* body mass index, *AHI* Apnoea-Hypopnoea Index

### Comparison between 63 patients with AS that completed home sleep-monitoring and 92 patients not fully completing

There was no significant difference in sex distribution, age, and BMI between the 63 patients that completed and the 92 patients who did not fully complete the home sleep-monitoring (Table 2). One of the 63 patients had been told by a doctor to have sleep apnoea and reported treatment for sleep apnoea, while 16 patients among those who were not assessed (1.6% vs 14.4%; *P* = 0.002) had been told to have sleep apnoea, out of which nine reported treatment.

**Table.2:** Clinical characteristics of 63 patients with ankylosing spondylitis (AS) assessed with home sleep-monitoring device and of 92 patients that not fully completed the examination

	AS sleep-monitoring *n* = 63	AS no sleep-monitoring *n* = 92	*P*-value
General characteristics			
Male sex, *n*	43 (68.2)	64 (69.6)	0.86
Age, years	55.4 (11.9)	55.6 (11.2)	0.92
Weight, kg	80.8 (14.8)	84.8 (21.5)	0.21
Length, cm	171.1 (9.1)	173.3 (9.6)	0.15
BMI, kg/m^2^	27.6 (4.4)	28.1 (5.9)	0.55
Ever smoker, *n*	31 (49.2)	40 (43.5)	0.48
Walk ≥ 10 min, days/week, *n*	4.5 (2.0, 7.0)*	5.0 (3.0, 7.0)*	0.39
AS-related variables			
Duration of symptoms, years	31.0 (11.2)	32.3 (12.4)	0.50
ESR, mm/h †	15.7 (14.4)	12.6 (9.4)	
	12.0 (5.0; 23.0)	10.0 (5.0; 18.8)	0.31
hsCRP, mg/L †	5.3 (7.4)	4.1 (4.9)	
	2.8 (1.1;7.0)	2.6 (0.9;5.0)	0.43
BASDAI, score	3.7 (1.7)	3.7 (2.0)	0.97
BASDAI fatigue, score	4.6 (2.2)	4.6 (2.6)	0.82
ASDAS-CRP, score	1.8 (0.7)	1.8 (0.7)	0.72
BASMI, score	4.2 (1.5)	4.1 (1.6)	0.63
BASFI, score	2.8 (1.7)	3.1 (2.2)	0.35
Chest expansion, cm	4.6 (1.9)	4.5 (1.9)	0.62
NSAID regular usage, *n*	35 (55.6)	60 (65.3)	0.23
csDMARD and/or bDMARD, *n*	17 (27.0)	21 (22.8)	0.55
≥ 1 syndesmophyte, *n*	34 (54.0)**	52 (56.5)	0.92
mSASSS, score	18.7 (21.2)	17.5 (20.5)	0.73
Comorbidities			
Asthma, *n*	7 (11.1)*	13 (14.1)	0.61
Diabetes, *n*	6 (9.5)	7 (7.6)	0.67
Metabolic syndrome, *n*	28 (44.4)	30 (32.6)	0.095
Hyperlipidemia, *n*	20 (30.3)*	21 (23.6)*	0.34
Sleep apnoea, *n*	1 (1.6)	16 (14.4)	0.002
ESS, score	6.6 (4.2)	7.1 (4.9)	0.51
Laboratory values			
LDL, mmol/L	3.4 (1.0)	3.2 (0.8)	0.19
HDL, mmol/L	1.5 (0.4)	1.6 (0.51)	0.44
Cholesterol, mmol/L	5.5 (1.2)	5.5 (1.0)	0.59

Values are mean (SD) or numbers of patients (%)

^†^ Values are also given as median (quartile 1, quartile 3)

Number of missing data: * = 1 ** = 2

*BMI* body mass index, *ESR* erythrocyte sedimentation rate, *hsCRP* high-sensitivity C-reactive protein, *BASDAI* Bath Ankylosing Disease Activity Index, *ASDAS* Ankylosing Spondylitis Disease Activity Score, *BASMI* Bath Ankylosing Spondylitis Metrology Index, *BASFI* Bath Ankylosing Spondylitis Functional Index, *NSAID* non-steroidal anti-inflammatory drug, *csDMARD* conventional synthetic disease modifying anti-rheumatic drug, *b* biologic, *mSASSS* Modified Stroke Ankylosing Spondylitis Score, *ESS* Epworth sleeping scale, *HDL* high-density lipoprotein, *LDL* low-density lipoprotein

### OSA in patients with AS

Twenty-five (39.7%) of the 63 patients with AS who fully completed the home sleep-monitoring had OSA and nine (14.3%) had OSAS. Twenty patients (31.7%) had mild OSA, five (7.9%) moderate, and none severe. The patients with OSA were significantly older, had a higher BMI and weight, had a longer duration of AS symptoms and more often had metabolic syndrome and ≥ 1 syndesmophyte, had a higher mSASSS, BASMI, BASFI, and ESS score, and lesser chest expansion, compared with patients without OSA (Table 3). Logistic regression analyses adjusted for sex and age revealed that higher BMI (OR 1.6, 95% CI 1.2–2.2), higher age (OR 1.1, 95% CI 1.0–1.2), and lesser chest expansion (OR 0.6, 95% CI 0.4–0.9) were determinants for OSA (Table 4). Supplementary Fig. 2 shows the correlation between AHI and age, r_s_ = 0.44, *P* =  < 0.001; AHI and BMI, r_s_ = 0.49, *P* =  < 0.001; and AHI and chest expansion r_s_ =  − 0.51, *P* < 0.001.

**Table.3:** Comparison between 25 patients with ankylosing spondylitis (AS) with obstructive sleep apnoea (OSA) and 38 patients with AS without OSA

	OSA *n* = 25	No OSA *n* = 38	*P*-value
General characteristics			
Male sex, *n*	15 (60.0)	28 (73.7)	0.25
Age, years	61.6 (7.8)	51.2 (12.4)	< 0.001
Length, cm	168.1 (8.4)	173.0 (9.1)	0.034
Weight, kg	85.6 (16.5)	77.7 (12.9)	0.038
BMI, kg/m^2^	30.3 (5.2)	25.8 (2.5)	< 0.001
Smoking ever, *n*	10 (40.0)	15 (39.5)	0.20
Walk ≥ 10 min, days/week, *n*	4.0 (2.0, 7.0)	5.0 (2.5, 7.0)*	0.96
Metabolic syndrome, *n*	16 (66.7)*	12 (31.6)	0.009
AS-related variables			
Duration of symptoms, years	34.8 (10.0)	28.4 (11.3)	0.023
ESR, mm/h †	15.7 (13.2)	15.7 (15.4)	
	9 (7.0; 23.5)	12 4.0; 22.3)	0.58
hsCRP, mg/L †	4.8 (4.4)	5.7 (8.9)	
	3.5 (1.5;7.0)	2.4 (0.6;8.3)	0.17
BASDAI, score	4.0 (1.7)	3.5 (1.8)	0.29
BASDAI fatigue, score	5.0 (2.0)	4.4 (2.3)	0.31
ASDAS-CRP, score	1.8 (0.6)	1.8 (0.7)	0.99
BASMI, score	5.0 (1.4)	3.7 (1.4)	0.001
BASFI, score	3.4 (1.9)	2.4 (1.5)	0.028
Chest expansion, cm	3.7 (1.5)	5.2 (1.8)	0.001
NSAID regular usage *n*	14 (56.0)	21 (55.2)	0.95
csDMARD and/or bDMARD, *n*	5 (20.0)	12 (31.6)	0.39
≥ 1 syndesmophyte, n	18 (75.0)*	16 (43.2)*	0.019
mSASSS, score	25.6 (24.5)	14.2 (17.7)	0.039
Laboratory values			
LDL, mmol/L	3.3 (0.8)	3.4 (1.2)	0.86
HDL, mmol/L	1.6 (0.4)	1.5 (0.4)	0.95
Cholesterol, mmol/L	5.6 (0.9)	5.5 (1.4)	0.69
ESS, score	8.1 (4.2)	5.6 (3.9)	0.018

Values are mean (SD) or numbers of patients (%)

^†^Values are also given as median (quartile 1, quartile 3)

Number of missing data: * = 1

*BMI* body mass index, *ESR* erythrocyte sedimentation rate, *hsCRP* high-sensitivity C-reactive protein, *BASDAI* Bath Ankylosing Disease Activity Index, *ASDAS* Ankylosing Spondylitis Disease Activity Score, *BASMI* Bath Ankylosing Spondylitis Metrology Index, *BASFI* Bath Ankylosing Spondylitis Functional Index, *NSAID* non-steroidal anti-inflammatory drug, csDMARD; conventional synthetic disease modifying anti-rheumatic drug, b; biologic, *mSASSS* Modified Stroke Ankylosing Spondylitis Score, *HDL* high-density lipoprotein, *LDL* low-density lipoprotein, *ESS* Epworth sleeping scale

**Table.4:** Univariable and age- and sex-adjusted logistic regression analyses with obstructive sleep apnoea (OSA) as dependent variable in 63 patients with ankylosing spondylitis (AS)

Variables	Odds ratio (95% CI) Univariable	*P*-value	Odds ratio (95% CI) Age- and sex-adjusted	*P*-value
Sex, male *n*	1.9 (0.6–5.5)	0.25	1.5 (0.4–4.8)	0.53
Age, years	1.1 (1.0–1.2)	0.002	1.1(1.0–1.2)	0.002
BMI, kg/m^2^	1.4 (1.1–1.7)	0.001	1.6 (1.2–2.2)	0.001
Duration of symptoms, years	1.1 (1.0–1.1)	0.028	1.0 (0.9–1.1)	0.79
BASMI, score	1.9 (1.3–2.9)	0.002	1.5 (0.9–2.5)	0.87
BASFI, score	1.4 (1.0–2.0)	0.038	1.3 (0.9–2.0)	0.88
Chest expansion, cm	0.6 (0.4–0.8)	0.002	0.6 (0.4–0.9)	0.007
≥ Syndesmophyte, *n*	3.9 (1.3–12.2)	0.017	3.0 (0.8–11.3)	0.1
mSASSS, score	1.0 (1.0–1.1)	0.047	1.0 (0.98–1.05)	0.25
Metabolic syndrome, *n*	4.3 (1.5–12.9)	0.008	1.4 (0.3–6.6)	0.69
ESS, score	1.2 (1.0–1.3)	0.023	1.2 (1.0–1.4)	0.29

*CI* confidence interval, *BMI* body mass index, *BASMI* Bath Ankylosing Spondylitis Metrology Index, *BASFI* Bath Ankylosing Spondylitis Functional Index, *mSASSS* Modified Stroke Ankylosing Spondylitis Score, *ESS* Epworth sleeping scale

## Discussion

In this study, we have investigated the prevalence of OSA and other measurements reflecting sleeping patterns assessed by a home sleep-monitoring device in a group of well-characterised patients with AS in comparison with matched controls. In addition, we have searched for AS-disease-related and non-disease-related factors associated with OSA in patients with AS. We found no difference in the prevalence of OSA between patients with AS and controls. About half of the AS patients and controls fulfilled the criteria of OSA. Furthermore, the most important determinants for OSA in patients with AS were higher age and BMI and lesser chest expansion, reflecting more severe AS disease.

A link between AS and OSA has been suggested, but knowledge has been limited. Prior small studies have reported a higher prevalence of OSA in patients with AS, in indirect comparisons with the prevalence in the general population [5, 6]. Furthermore, an elevated prevalence of sleep apnoea was reported in AS patients compared with controls in a large US administrative claims database [1]. In contrast, we did not find a higher prevalence of OSA in AS patients in the current controlled study. The discrepancy between our and previous findings might be explained by the disparate methods used in these studies, such as different ways of recruiting the patients, diverse devices to assess OSA, and indirect comparisons with controls versus direct comparison in our study.

Concerning the non-AS-disease-related factors, we found, in the adjusted logistic regression analyses, that OSA was related to higher BMI and age, in line with findings in the general population [21, 22]. Regarding the AS-related characteristics, various factors were related to the presence of OSA in the non-adjusted logistic regression analyses, while most of the associations, except lesser chest expansion, disappeared when adjusting for age and sex. Not surprisingly, we showed a significantly higher ESS-score among the AS patients with OSA compared to those without OSA. This suggests that ESS may be used as a screening tool for OSA in patients with AS.

The latest guidelines from the American Academy of Sleep Medicine (AASM) recommend treatment for sleep apnoea in patients with AHI ≥ 15 events/h and/or AHI ≥ 5 events/h with symptoms [12]. Thus, according to the AASM guidelines, 13/63 (20.6%) of the investigated patients with AS qualified for treatment. For biological DMARDs, the literature shows conflicting results about the association between TNF-inhibitors and OSA, one study found a lower frequency of OSA among TNF-inhibitor-treated patients, while two other studies did not, in line with our findings [23–25].

There are some limitations to be acknowledged. Only 63 (40.6%) of 155 patients with AS were completely assessed with the home sleep-monitoring device, which means that one may not draw too strong conclusions in sub-group analyses. The golden standard, polysomnography for diagnosing sleeping disorders, was not performed, but a simplified home sleep-monitoring device was used. The strengths of this study are the use of a matched control group, the systematic use of the same validated device [20] in both the patients and the controls, and the very well-characterised patients with AS. Although only 40.6% of the AS patients completed the assessment, the 63 examined patients did not differ in any clinical characteristics compared to the 92 patients not examined with the device. Furthermore, the 46 patients with AS who were compared with the matched controls from SCAPIS did not differ in AS-related characteristics from the 109 patients with AS that were not compared with the SCAPIS controls. Thus, we believe that the studied patients with AS quite well represent patients with AS followed at a university hospital in northern Sweden.

In conclusion, the prevalence of OSA in patients with AS was not higher than in matched controls. The AS patients with OSA had more daytime sleepiness, had a higher BMI, were older, and, importantly, had also lesser chest expansion, which reflects a more severe AS disease, compared with patients without OSA. These are the most important factors to consider when trying to identify OSA in patients with AS.

## Supplementary Information

Below is the link to the electronic supplementary material.

Supplementary file1 (DOCX 199 KB)

## Data Availability

The data sets analysed during the current study are not publicly available due to the Swedish legislation (the Personal Data Act), but a limited and fully anonymised data set that supports the main analyses is available from the corresponding author on request.
