# Supplementary material for: Prevalence and factors related to sleep apnoea in ankylosing spondylitis
Source: Clin Rheumatol. 2021 Sep 28;41(2):491–8. doi: 10.1007/s10067-021-05924-z (PMC8782774; doi:10.1007/s10067-021-05924-z)
Supplement: Supplementary file 1 — Supplementary file1 (DOCX 199 KB) [file 10067_2021_5924_MOESM1_ESM.docx]

**Journal Name**:

Clinical Rheumatology

**Title:**

Prevalence and factors related to sleep apnoea in ankylosing spondylitis

**Manuscript type:**

Brief Report

**Authors:**

Adrian Wiginder, Carin Sahlin-Ingridsson, Mats Geijer, Anders Blomberg, Karl A Franklin, Helena Forsblad-d’Elia

**Corresponding author:**

Helena Forsblad-d’Elia

E-mail: helena.forsblad@rheuma.gu.se

**Supplementary table 1.** *Comparison between the 46 patients with ankylosing spondylitis (AS) that the matching was based on and the other 109 patients.*

|  | AS matched  n=46 | AS not matched  n=109 | P-value |
| --- | --- | --- | --- |
| **General characteristics** |  |  |  |
| Male sex, n | 30 (65.5) | 77 (70.6) | 0.57 |
| Age, years | 57.2 (7.5) | 54.7 (12.8) | 0.22 |
| Weight, kg | 81.1 (15.4) | 84.0 (20.4) | 0.38 |
| Length, cm | 169.8 (8.6) | 173.5 (9.6) | 0.024 |
| BMI, kg/m^2^ | 28.1 (4.8) | 27.8 (5.6) | 0.73 |
| Smoking ever, n | 24 (52.2) | 47 (43.1) | 0.38 |
| Walk ≥ 10 min, days/week, n | 4.0 (1.0, 7.0) | 5.0 (3.0, 7.0)**** | 0.085 |
| Metabolic syndrome, n | 25 (55.6)* | 32 (29.4)* | 0.003 |
| **AS-related variables** |  |  |  |
| Duration of symptoms, years | 32.6 (9.3) | 31.4 (12.9) | 0.57 |
| ESR, mm/h | 17.0 (15.7)  12.5 (7.8;23.3) | 12.5 (9.5)  10.0 (5.0;19.0) | 0.14 |
| hsCRP, mg/L | 5.9 (8.3)  2.9 (1.5;7.0) | 4.1 (4.8)  2.3 (0.8;5.5) | 0.12 |
| BASDAI fatigue, score | 5.0 (2.2) | 1.8 (0.7) | 0.68 |
| BASDAI, score | 3.8 (1.8) | 3.7 (2.0) | 0.72 |
| ASDAS-CRP, score | 1.9 (0.7) | 1.8 (0.7) | 0.50 |
| BASMI, score | 4.4 (1.4) | 4.0 (1.6) | 0.13 |
| BASFI, score | 3.0 (1.8) | 3.0 (2.1) | 0.95 |
| Chest expansion, cm | 4.3 (1.7) | 4.6 (2.0) | 0.28 |
| mSASSS, score | 21.9 (22.6) | 16.4 (19.8) | 0.63 |
| NSAID regular usage, n | 24 (52.2) | 71 (65.1) | 0.15 |
| csDMARD and/or bDMARD, n | 14 (30.4) | 24 (22.0) | 0.31 |
| ≥ 1 syndesmophyte, n | 27 (58.7)** | 59 (54.1) | 0.47 |
| **Laboratory values** |  |  |  |
| LDL, mmol/L | 3.4 (1.1) | 3.2 (0.8) | 0.08 |
| HDL, mmol/L | 1.5 (0.5) | 1.6 (0.5) | 0.55 |
| Cholesterol, mmol/L | 5.6 (1.3) | 5.4 (1.0) | 0.17 |
| **ESS, score** | 6.8 (4.1) | 6.9 (4.8) | 0.96 |

*Values are mean (SD) or numbers of patients (%).*

*Number of missing data: *=1 **=2*

*BMI; body mass index, ESR; erythrocyte sedimentation rate, hsCRP; high-sensitiviy C-reactive protein, BASDAI; Bath Ankylosing Disease Activity Index, ASDAS; Ankylosing Spondylitis Disease, Activity Score, BASMI; Bath Ankylosing Spondylitis Metrology Index BASFI; Bath Ankylosing Spondylitis Functional Index, NSAID; non-steroidal anti-inflammatory drug, csDMARD; conventional synthetic disease modifying anti-rheumatic drug, b; biologic, mSASSS; Modified Stroke Ankylosing Spondylitis Score, HDL; High Density Lipoprotein, LDL; Low Density Lipoprotein, ESS; Epworth Sleeping Scale.*

**Supplementary Fig 1.** Procedure for inclusion of patients with ankylosing spondylitis (AS) and control persons from the Swedish CardioPulmonary bioImage Study (SCAPIS)

Footnote: BMI; body mass index


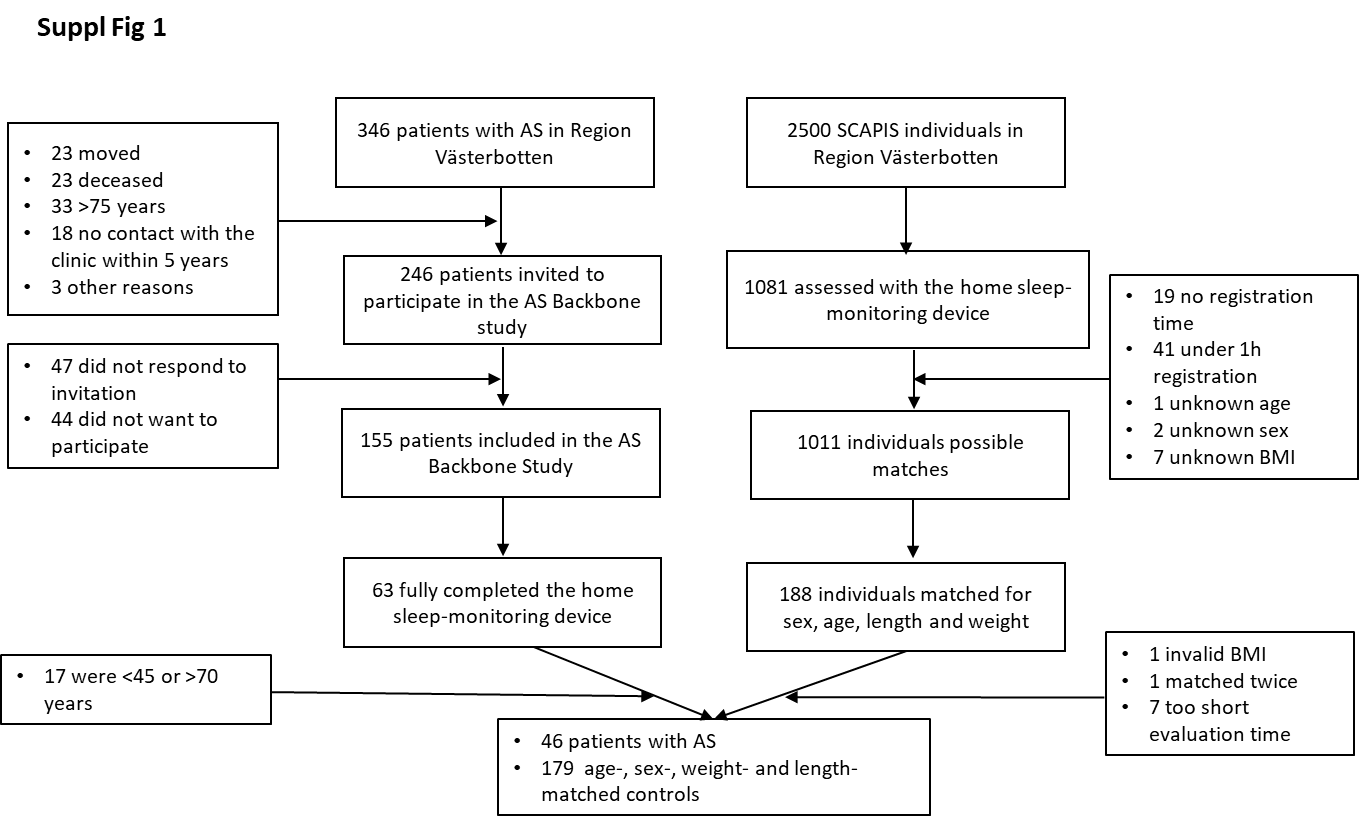


**Supplementary Fig 2.** Scatterplots between Apnoea-Hypopnoea Index (AHI) and a) age, b) body mass index (BMI), and c) chest expansion in 63 patients with ankylosing spondylitis

*
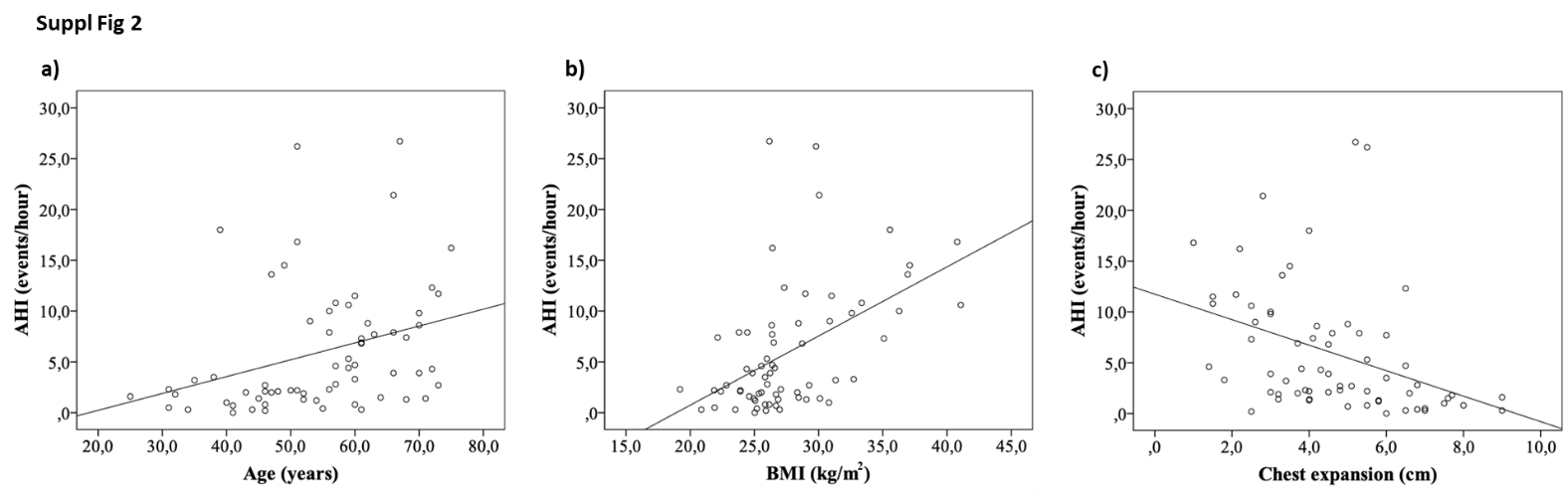
*
